# Supplementary figures and images for: Discovering individual-specific gait signatures from data-driven models of neuromechanical dynamics
Source: PLoS Comput Biol. 2023 Oct 27;19(10):e1011556. doi: 10.1371/journal.pcbi.1011556 (PMC10610102; doi:10.1371/journal.pcbi.1011556)

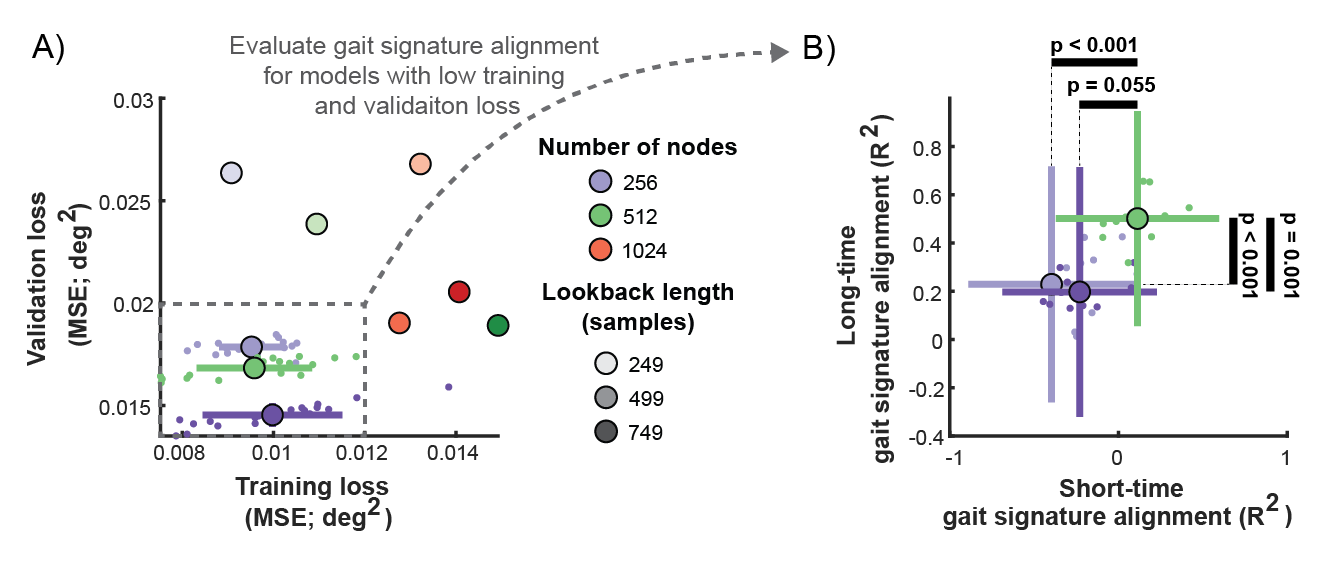

Supplement: S1 Fig — In both plots, small dots represent the average values across trials for each random initialization of each model. Large dots and bars denote the average and standard deviation of model performance metrics across initializations. Left: Training and validation loss (RMSE) for all 12 hyperparameter pairs. Models in the lower-left consider are considered better. Right: Long- and short-time prediction performance (R2) for the 3 hyperparameter pairs with the lowest training and validation loss. Models in the upper-right corner are considered better. (TIF) [file pcbi.1011556.s009.tif]

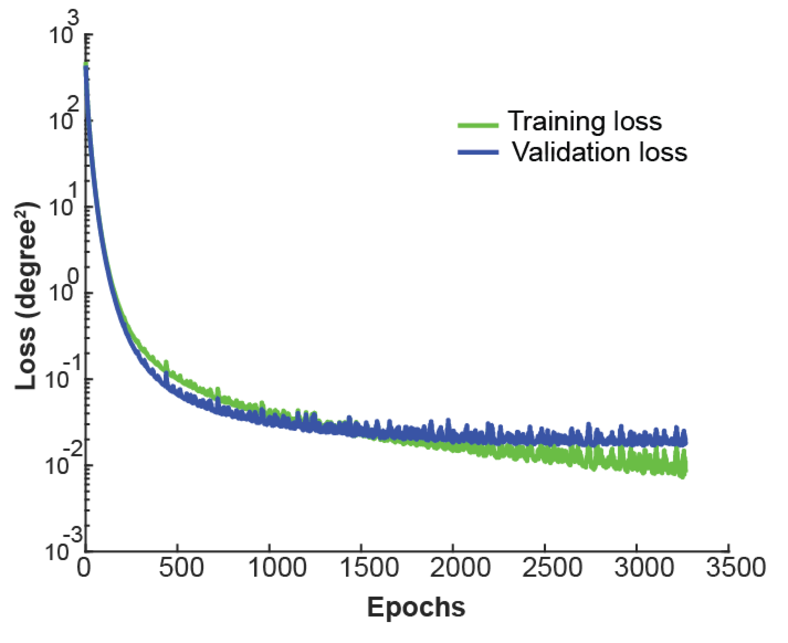

Supplement: S2 Fig — (TIF) [file pcbi.1011556.s010.tif]

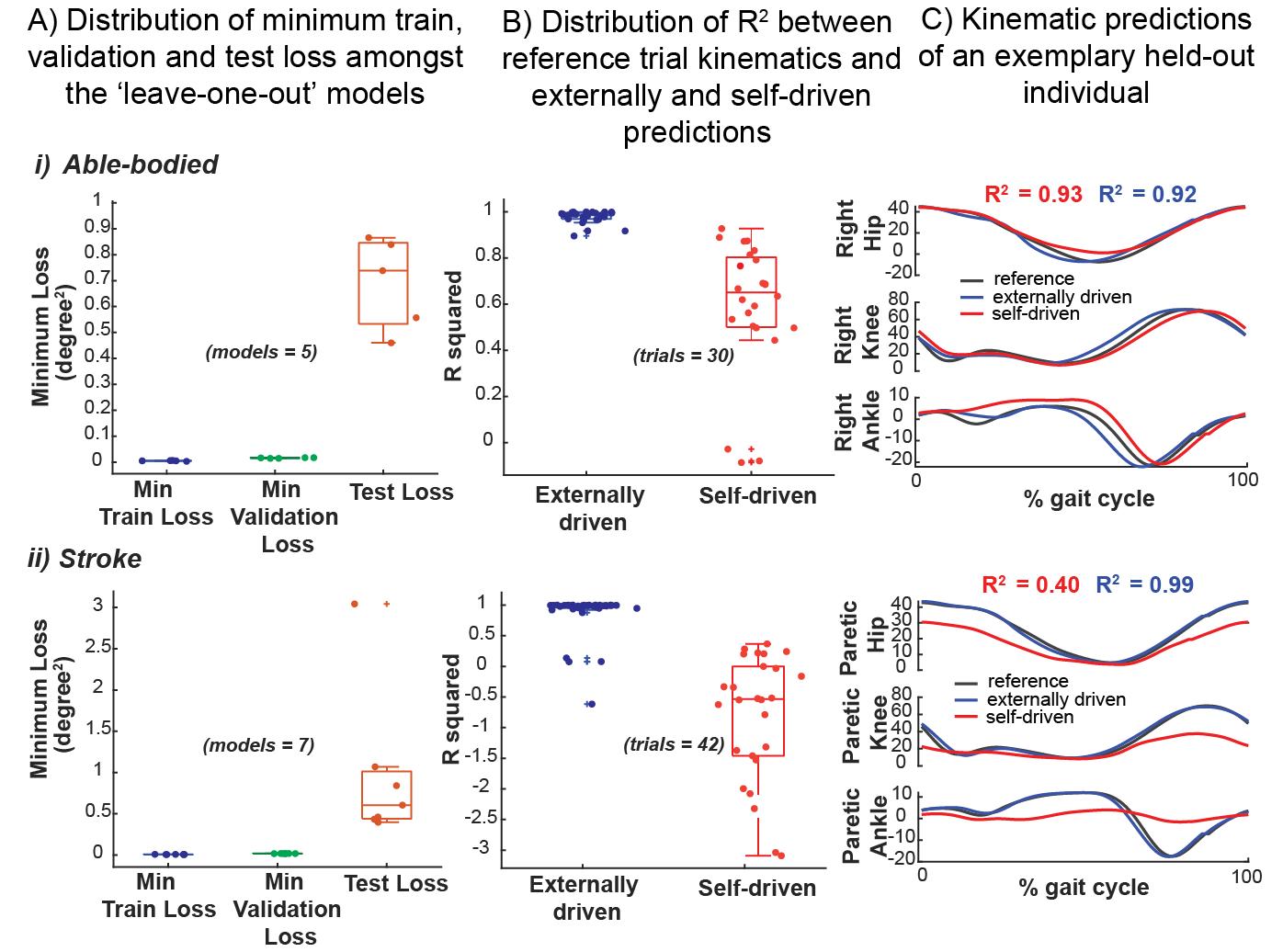

Supplement: S3 Fig — The minimum train loss (blue) and validation loss (green) was low (< 0.02 degrees2) for 5 models that were trained each with a single able-bodied individual held out of the training data (A, i) and 7 models each with a stroke individual held out of training data (A, ii). The magnitude and range of the test loss (evaluation of model on the held-out data) (orange) was higher than the respective minimum training and validation losses for both held-out able-bodied (A, i) and stroke (A, ii) models. The magnitude and range of test losses evaluated on held-out able-bodied individuals, however, were lower than models evaluated on held-out stroke data. The models generate external predictions (blue) of held-out test trials with higher R2 values than that of self-driven predictions (red) in models evaluated on both able-bodied (B, i) and stroke trials (B, ii). The models can generate external kinematic predictions (blue) of held-out able-bodied (B, i) trials better than that of stroke (B, ii). Self-driven predictions of stroke kinematics were generally very low (R2 values below 0.5). Models were incapable of generating self-driven predictions for 5 of 30 able-bodied trials and 16 of 42 stroke trials. (C) shows reference (black), externally driven (blue) and self-driven (red) phase averaged kinematic predictions for an exemplary able-bodied trial (C, i) and exemplary stroke trial (C, ii). Models can predict kinematics of held-out able-bodied trials better (higher R2) than that of stroke. (TIF) [file pcbi.1011556.s011.tif]

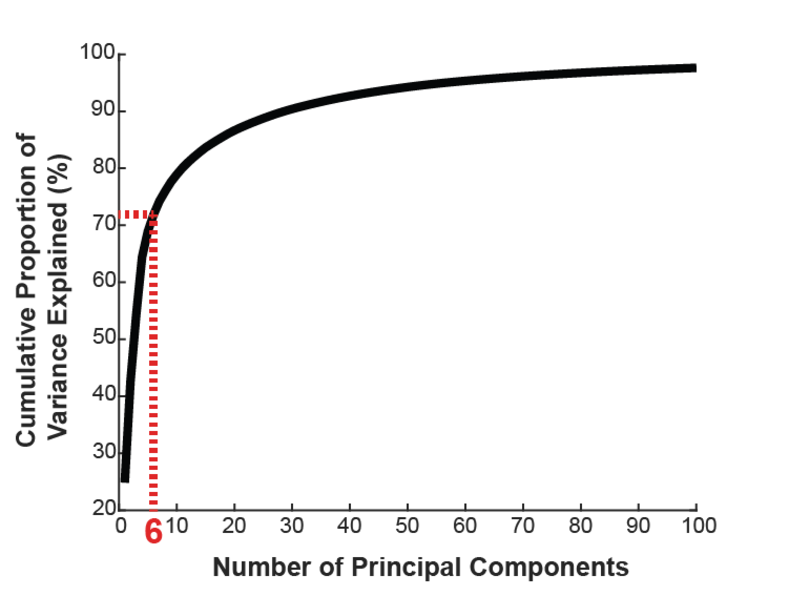

Supplement: S4 Fig — Six (6) principal components (PCs) explained 77% of the variance in the gait dynamics. The top 6 dominant PCs were used to develop the gait signature. (TIF) [file pcbi.1011556.s012.tif]

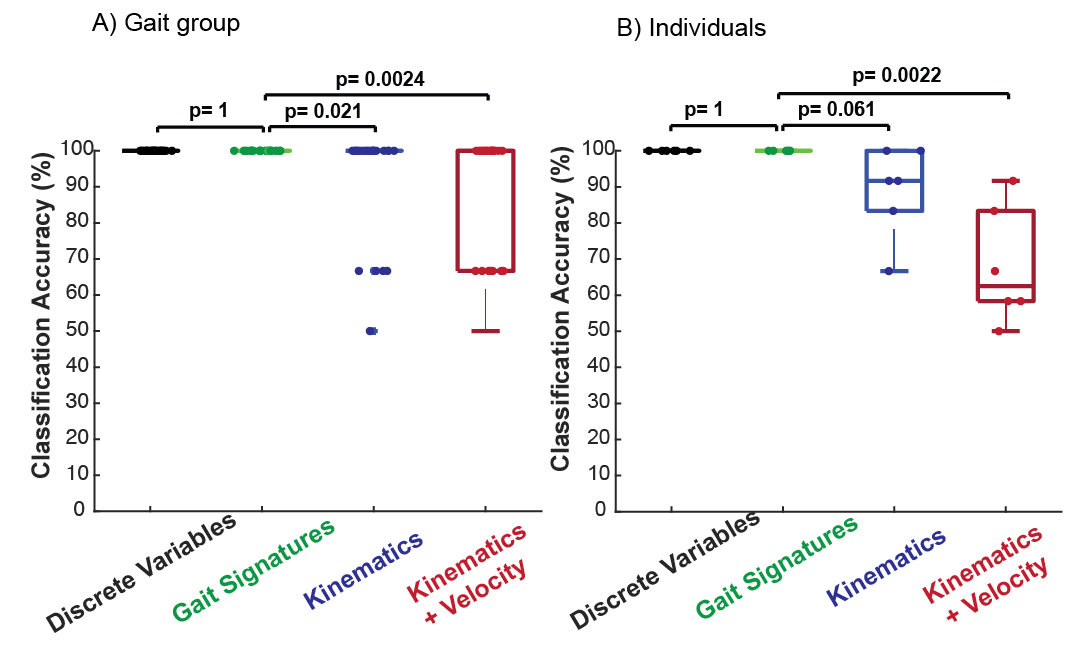

Supplement: S5 Fig — Using k = 25 folds, RNN gait signatures distinguished between impaired and unimpaired gait with 100% accuracy, along with the 26 discrete variables (100%, p = 1), whereas kinematic (92.67 ± 0.15%, p < 0.05) and kinematics & velocity (88.67 ± 0.17%, p <0.05) discrimination were significantly lower. Using k = 6 folds, SVM classification of individuals was most accurate using RNN gait signatures and discrete variables (100%), lower using kinematics (88.9 ± 0.13%, p = 0.061) and significantly lower using a kinematics and velocity (68.10 ± 0.16%, p < 0.05). (TIF) [file pcbi.1011556.s013.tif]

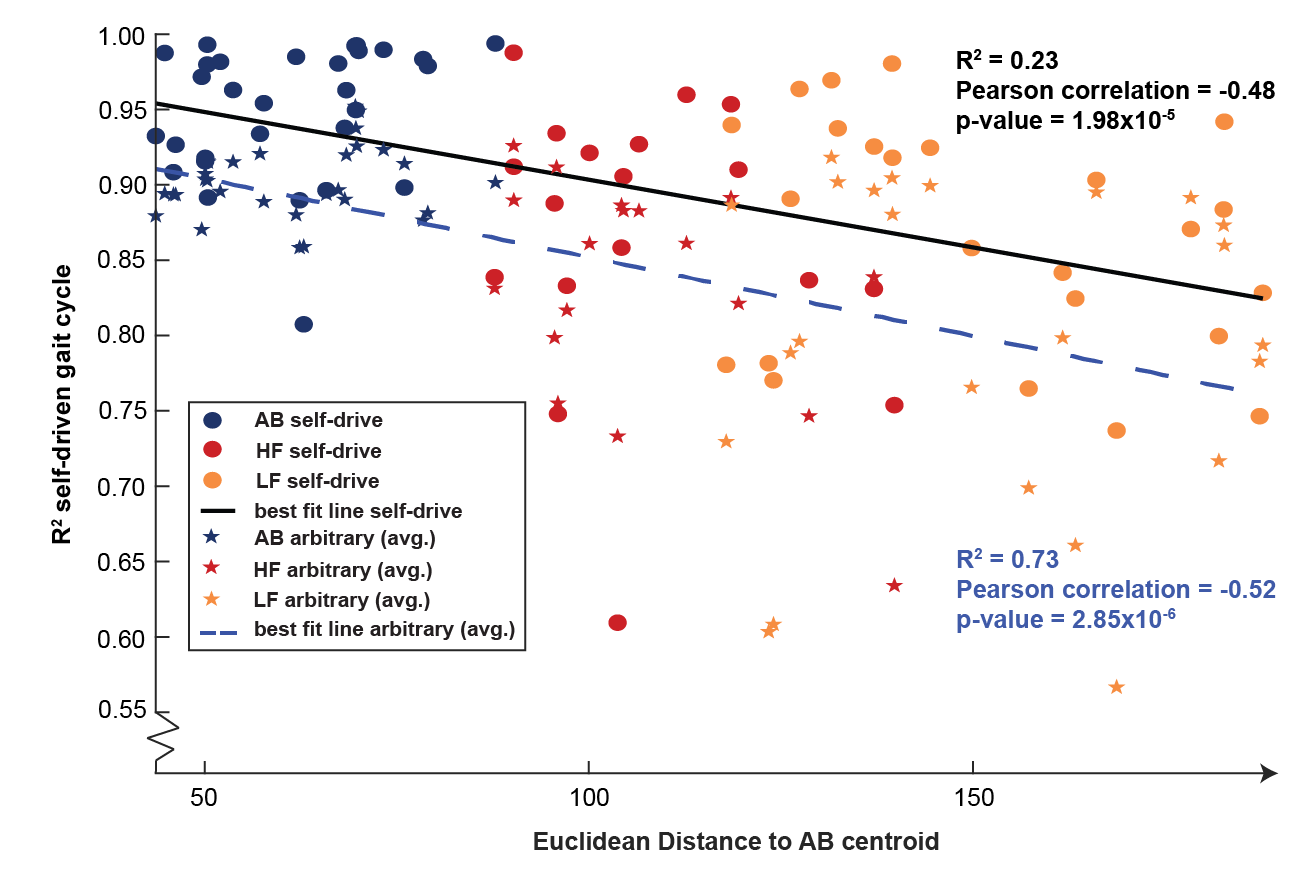

Supplement: S6 Fig — There exists a negative correlation between self-driven R2 and Euclidean distance to the AB centroid that is statistically significant at the 0.05 level (S6 Fig, dots). Lower functioning stroke survivors are located further away from the able-bodied centroid, however remarkably all but one (outlier) of the R2 values are above 0.73 in both high and lower-functioning individuals. Even though the model can better predict able-bodied future kinematics better than stroke (R2 values above 0.8), the ability of our model to predict at least a single gait cycle of future stroke kinematics with R2 above 0.73 is promising. To provide a control for this analysis, we found the average R2 values between the self-driven prediction of the last full gait cycle of each trial and 5 randomly selected gait cycles from other randomly selected individuals in the same gait group (able-bodied vs. stroke) (S6 Fig, stars). This control would effectively reveal whether our model learned each individual’s specific gait pattern or was just producing arbitrary (averaged) gait patterns. We expect that if the model learned individuals specific gait patterns, then the distribution of the R2 values of the self-driven kinematic predictions would be significantly different to that of the averaged R2 values corresponding to arbitrary gait patterns. In fact, the averaged R2 of the generic gait cycle comparisons ranged from 0.56 to 0.95 (compared to 0.61–0.99 in self-driven predictions). Further, the distributions of the R2 outputs for both able-bodied and stroke individuals were significantly different (at the 0.05 level) to self-driven R2 outputs with p-values 5.5x10^-6^ and 5.4x10^-3^ respectively using the Wilcoxon Rank-Sum test. This reveals that even for stroke survivors the model has learned their dynamics in some capacity and the model isn’t simply predicting arbitrary gait patterns. (TIF) [file pcbi.1011556.s014.tif]

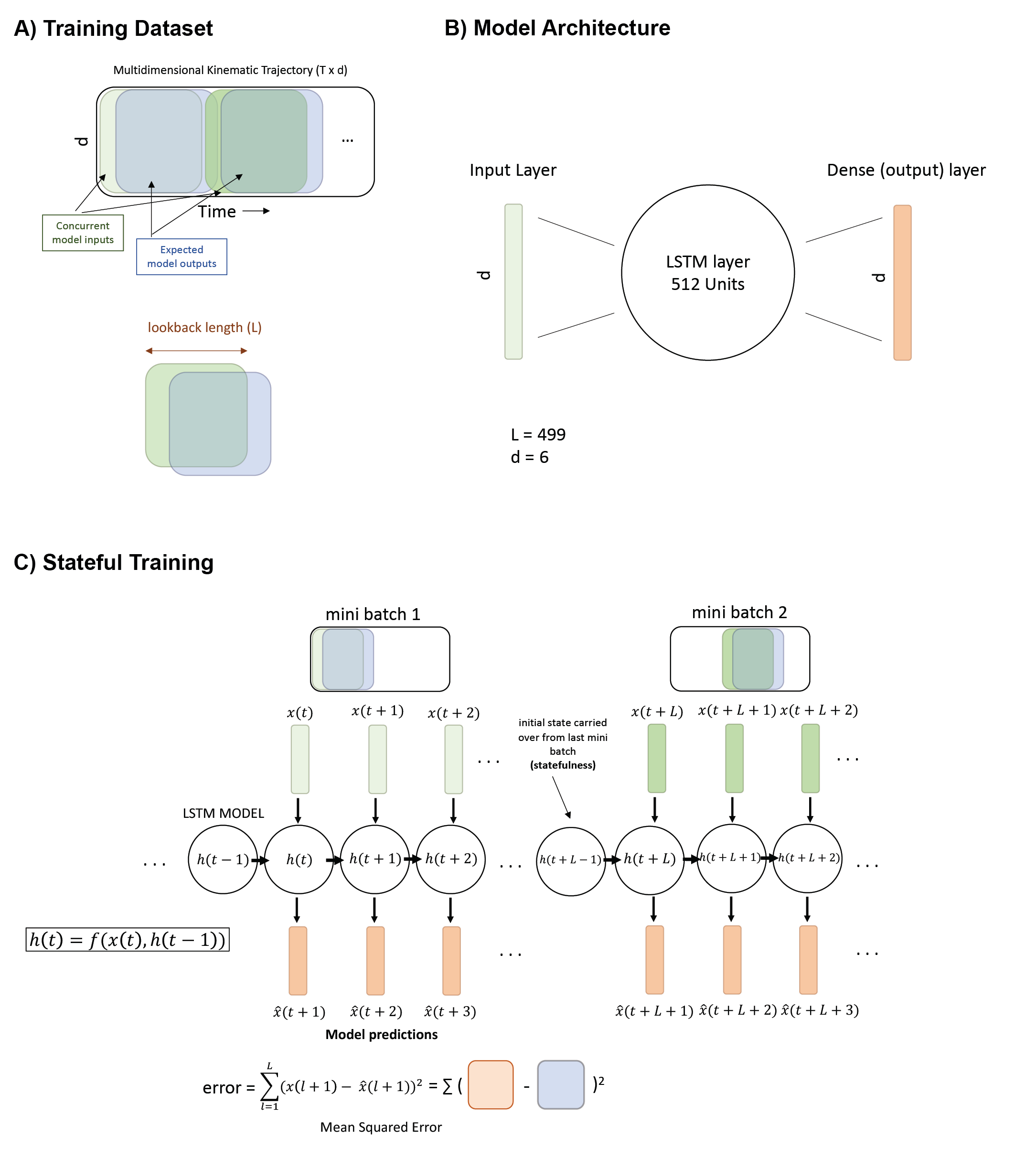

Supplement: S7 Fig — A) Training dataset: Using the 6-dimensional gait trajectory, the inputs (green) were concurrent segments from the gait trajectory, each one 499-time steps long. The outputs (blue) were 1-shifted (in time) segments of inputs. B) Model architecture: Our model consisted of an input layer, a hidden layer composed of 512 LSTM units, and a 6-unit Dense output layer. C) Stateful training: The hidden state of an RNN at time t is a function of the input at time t and the hidden state at time t-1. The model starts with processing the first mini-batch, calculating a new hidden state at each t and predicting gait kinematics at time t+1 given kinematics data at t. At the end of the mini-batch processing, the model calculates MSE over the entire mini-batch to calculate error for backpropagation and to update model weights. The final hidden state h(t+L) is used as the initial hidden state for generating predictions for the next mini batch (L is the temporal length of each mini-batch). The hidden state is initialized as zero before processing the first mini-batch. (TIF) [file pcbi.1011556.s015.tif]

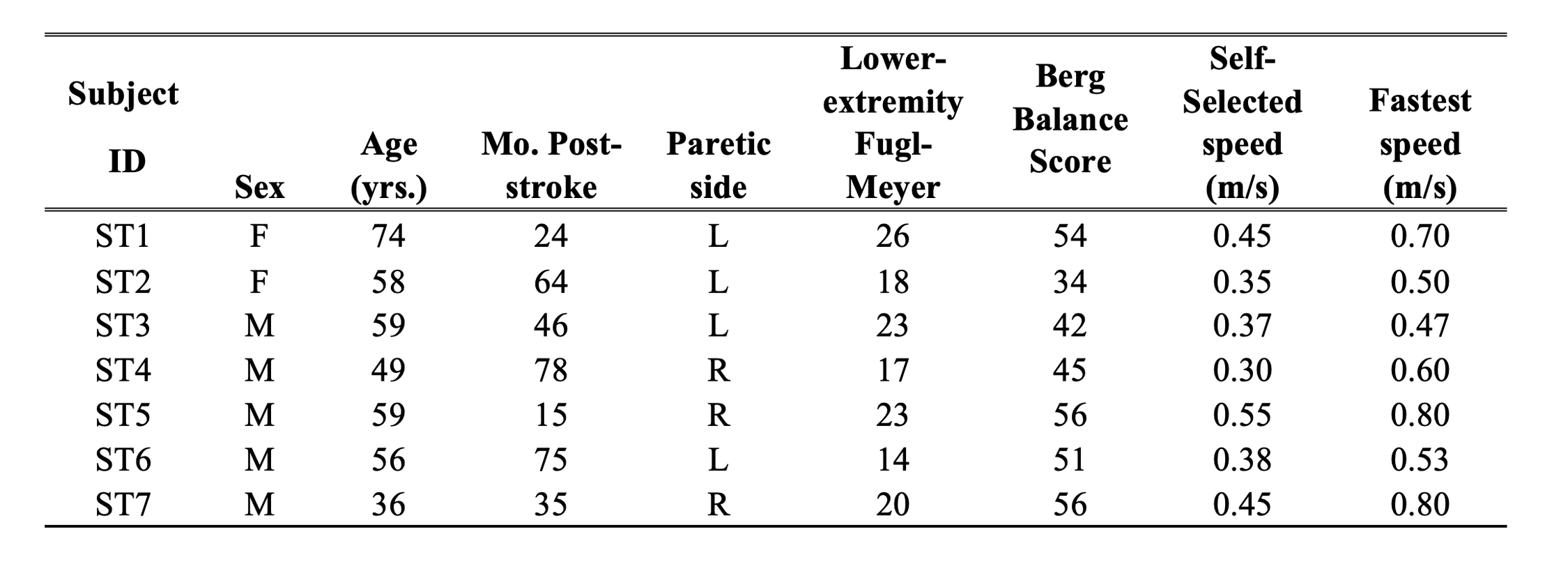

Supplement: S1 Table — (TIF) [file pcbi.1011556.s016.tif]
