# Supplementary material for: Discovering individual-specific gait signatures from data-driven models of neuromechanical dynamics
Source: PLoS Comput Biol. 2023 Oct 27;19(10):e1011556. doi: 10.1371/journal.pcbi.1011556 (PMC10610102; doi:10.1371/journal.pcbi.1011556)
Supplement: Attachment — Submitted filename: Response to reviewers2.docx [file pcbi.1011556.s017.docx]

**Response to Reviewers**

We thank the reviewers for their dedicated time and invaluable insights provided in the evaluation of our recently submitted manuscript. We are honored that the reviewers found the work “exciting”, “significant”, “well-written”, “clear” and “precise”. We appreciate that the reviewers acknowledge the relevance of this work to clinical researchers interested in locomotion patterns. The thorough and constructive feedback received from the reviewers has been instrumental in enhancing the quality and rigor of our manuscript. Their critical analysis has guided us in refining our research through providing additional analyses and figures, strengthening (or softening where necessary) our arguments, and clarifying intricate points, ultimately contributing to a more thorough and clearer manuscript. We truly appreciate their commitment to scholarly excellence and helping elevate the impact of our research. Our pointwise responses to reviewer comments, and actions taken to improve the revised manuscript are listed in detail below.

**Reviewer #1**: This is an exciting work on investigating and interpreting the neuromechanical dynamics accountable for distinct gait patterns using a machine learning framework, particularly recurrent neural networks (RNNs). The authors collected joint kinematics using motion capture from 12 adults (5 able-bodies and 7 stroke survivors) walking at different speeds (0.3-1.8m/s). The study found that the RNN model could detect individual gait signatures that were consistent across various walking speeds, with stroke survivors showing greater gait variations at slower speeds. Although the contributions of the work are significant and relevant to the clinical researchers in examining animal locomotion patterns, there are a few concerns, as follows, that question the technical soundness of the work.

1. Referring Figure 1(b) and 2(b), the authors claim that the cluster of low functioning stroke survivors at self-selected speed<0.4m/s is at farthest distance from the able-bodied cluster. However, it is quite evident from the results that encapsulated cluster of low functioning stroke has several outliers, i.e., self-selected speed>0.4 m/s (bigger circles). The authors are suggested to justify the reasoning behind ignoring the effect of such outliers.

Thank you for the comment. We agree with the reviewer that the low-functioning group may not form an obvious distinct cluster. Note that low-functioning stroke survivors were only characterized based on the clinical definition of having a self-selected walking speed of <0.4 m/s. However, although gait speed is limited in its measurement properties and discriminative ability, gait speed is widely used in research to categorize walking function in people with stroke and other neurological impairments. We did not use any information from a clustering algorithm to characterize low- vs high-functioning individuals. The encircled low-functioning stroke survivors in Figure 2 B were labeled post-hoc as a way to demonstrate the lack of a single cluster characterizing low- vs high-functioning stroke survivors. We acknowledge that the reviewer is correct in stating that some low-functioning subjects faster speeds were > 0.4m/s. Please note, the sizes of the circles are relative to each individual. i.e., an individual’s slowest speed is represented by the smallest circle, and this specific size is consistent with all the slowest speeds across subjects (although the actual value of the slowest speeds between individuals differs). Given the clarification above, we do not believe we have ignored any outliers. Moreover, these results in fact highlight the overall strength of our approach, as the gait signatures are more individual- rather than speed- specific, and the fact that the low-functioning individuals have more inter-individual variability in this representation is precisely what we would expect, as there are many more ways to walk atypically than there are ways to walk typically. To improve the clarity of this result, we have updated Figure 2 to encapsulate both the low and higher functioning stroke survivors as well and clearly label each imposed categorization.

We also provide a clearer description of this figure and discussion of its interpretation to prevent confusion. The updated text are as follows:

Lines 293-297: “We calculated the Euclidean distance between individuals’ self-selected speed trial gait signature and the calculated able-bodied centroid (Fig 2B, i black square) and the results shown on the plot to the right reveal that low-functioning stroke survivors (characterized based on the clinical definition of having a self-selected walking speed of <0.4 m/s) are further away from the able-bodied cluster than the high-functioning stroke survivors.”

Lines 297-301: “Note that no information from a clustering algorithm was used to characterize low- vs high-functioning individuals. The encircled low-functioning stroke survivors (Fig 2B, i, orange enclosure) were labeled post-hoc to demonstrate the lack of a single cluster characterizing low- vs high-functioning stroke survivors.”

2. Given Figure 5, the generalization capability of the trained RNN model is checked by interpolating gait kinematics across speeds, taking 1.32 m/s and 0.43 m/s. Although both speeds are unseen; however, within the slow and fast speed limit. The generalization capability will be more evident if the unseen speed will be outside the training range.

Thank you for the comment. Although we would like to have a model capable of generalizing speeds beyond the ranges in the input data, it is unlikely that an RNN model could achieve this goal. RNNs in general are highly dependent on the training data it sees to learn patterns in the dataset and thus are typically not capable of extrapolating. Thus, we do not expect it to be able to generalize to speeds outside of the speed range of data provided in training. The demonstrated generalization across speeds shows that the RNN can predict intermediate speeds even when the kinematics do not progress linearly. A benefit of this capability is that data that deviations from the walking speeds in the training set can still be analyzed, and it may reduce the number of speeds required in the training set to achieve a model that is valid across a given range of speeds. To address this comment, we modified the Results section lines 469-477 to acknowledge these limitations of the model and highlight specifically the benefit of this capability.

Lines 469-477: “We acknowledge that our model likely is not capable of generalizing to speeds beyond the ranges of the input data (extrapolating), as RNNs are highly dependent on the training data that it sees to learn patterns in the dataset. One benefit of this capability, however, is that any data that deviates from the walking speeds in the training set can still be analyzed, reducing the number of speeds required in the training set to achieve a model that is valid across a range of speeds. Additionally, our small sample size limits the amount of data the RNN sees for each diverse type of stroke dynamics, thus, with a larger sample size of stroke survivors and longer trials, the RNN may be able to make better kinematic predictions of lower-functioning stroke survivors.”

We also added statements in the Discussion Lines 780-781 referring to this point.

Lines 780-781: “Furthermore, our model is likely only capable of generalizing to speeds within the speed ranges of the input data.”

3. It is quite obvious that the RNN model would able to generalize able body kinematics much better than stroke survivors due to the significant difference in impaired gait kinematics among the stroke survivors. In that case, given S3 Figure, two questions must be investigated:

(a) How well the RNN model generalizes for the high functioning stroke survivors? Is the self-driven R^2 increasing due to closer cluster distance to centroid of able-bodied individuals?

Thank you for your comment. We agree with the reviewer that the model generalizes to able-bodied kinematics better than that of stroke survivors. These differences may be attributed to the limited sampling of individuals across the range of post-stroke impairments in our dataset. While the tight grouping of able-bodied participants may enable the model to better learn the dynamics of gait of able-bodied individuals through interpolation, it is likely that we lack sufficient data to enable prediction of gait deficits in stroke survivors because the heterogeneity of gait deficits makes interpolating between individuals difficult. Perhaps with a larger dataset and overlapping impairments, the model would be better able to generalize to stroke individuals. This emphasizes the need to leverage the current work and apply this approach to other larger-sample gait datasets collected using diverse walking conditions, which is one of our long-term goals, and is now emphasized in the Discussion and Future Directions.

To address comment 3(a), we have now added a new analysis and supplementary figure (S6 Figure) to demonstrate the relationship between the R2 values of the RNN self-driving predictions and the gait signature distance to the able-bodied centroid (AB). There exists a negative correlation between self-driven R^2^ and Euclidean distance to the AB centroid that is statistically significant at the 0.05 level (S6 Figure, dots). Lower functioning stroke survivors are located further away from the able-bodied centroid, however remarkably all but one (outlier) of the R^2^ values are above 0.73 in both high and lower-functioning individuals. Even though the model can better predict able-bodied future kinematics better than stroke (R^2^ values above 0.8), the ability of our model to predict at least a single gait cycle of future stroke kinematics with R^2^ above 0.73 is promising. To provide a control for this analysis, we found the average R^2^ values between the self-driven prediction of the last full gait cycle of each trial and 5 randomly selected gait cycles from other randomly selected individuals in the same gait group (able-bodied vs. stroke) (S6 Figure, stars). This control would effectively reveal whether our model learned each individual's specific gait pattern or was just producing arbitrary (averaged) gait patterns. We expect that if the model learned individuals specific gait patterns, then the distribution of the R^2^ values of the self-driven kinematic predictions would be significantly different to that of the averaged R^2^ values corresponding to arbitrary gait patterns. In fact, the averaged R^2^ of the generic gait cycle comparisons ranged from 0.56 to 0.95 (compared to 0.61 to 0.99 in self-driven predictions). Further, the distributions of the R^2^ outputs for both able-bodied and stroke individuals were significantly different (at the 0.05 level) to self-driven R^2^ outputs with p-values 5.5x10^-6^ and 5.4x10^-3^ respectively using the Wilcoxon Rank-Sum test. This reveals that even for stroke survivors the model has learned their dynamics in some capacity and the model isn’t simply predicting arbitrary gait patterns.

**S6 Figure: Relationship between participants’ similarity to able-bodied gait signatures and the RNN’s ability to predict gait kinematics over one gait cycle.** There exists a negative correlation between self-driven R^2^ and Euclidean distance to the AB centroid that is statistically significant at the 0.05 level (S6 Figure, dots). The average R^2^ values between the self-driven prediction of the last full gait cycle of each trial and 5 randomly selected gait cycles from other randomly selected individuals in the same gait group (able-bodied vs. stroke) served as the control (S6 Figure, stars). This control would effectively reveal whether our model learned each individual's specific gait pattern or was just producing arbitrary (averaged) gait patterns. The averaged R^2^ of the generic gait cycle comparisons ranged from 0.56 to 0.95 (compared to 0.61 to 0.99 in self-driven predictions). Further, the distributions of the R2 outputs for both able-bodied and stroke individuals were significantly different (at the 0.05 level) to self-driven R2 outputs with p-values 5.5x10-6 and 5.4x10-3 respectively using the Wilcoxon Rank-Sum test. This reveals that even for stroke survivors the model has learned their dynamics in some capacity and the model isn’t simply predicting arbitrary gait patterns.

We have added the following text to the Discussion Lines 781-788: “There was a moderate association between participants' similarity to able-bodied gait signatures (distance to the able-bodied centroid) and the RNN's ability to predict gait kinematics over one gait cycle (S6 Figure). This association is likely due to the RNN favoring able-bodied dynamics during model fitting, which were more homogeneous than those of high- or low-functioning stroke survivors. A larger post-stroke sample in future applications of our approach may improve the RNN's ability to encode and predict pathological gait dynamics. Further, the reduced predictive power for the stroke participants can be attributed to our model architecture’s relative simplicity and short time series (15 seconds/ 1500 sample points per trial).”

(b) How one can establish a relation between the differences in joint kinematics (varying from high to low functioning stroke survivors) and impaired anthropometric parameters such as flexion/extension deformities.

Our current RNN model does not include anthropometrics parameters as an input variable and is not currently designed to account for differences in joint kinematics due to neural versus biomechanical constraints. To address this concern, we now clarify in the revised Results lines 198-201 that the model input parameters do not include any anthropometric or clinical characteristics. Furthermore, the study inclusion criteria for post-stroke participants would have excluded severe contractures or deformities that could interfere with normal ambulation. We also agree with the reviewer’s point and note in our Discussion lines 746-754 that findings from our RNN model would benefit from being interpreted and correlated with clinical evaluation. The RNN model can form a valuable adjunct to conventional clinical methods and is not intended to replace them.

Lines 198-201: “Our model input parameters only include kinematic data and do not include anthropometric information or clinical characteristics and do not account for differences in joint kinematics due to neural versus biomechanical constraints.”

Lines 746-754: “Our study inclusion criteria exclude severe contractures or deformities that interfere with normal ambulation and in future work the gait signatures would need to be interpreted and correlated with clinical evaluation of strength, range of motion, sensorimotor impairment, and/or limb deformities. The demographic and clinical information of the stroke participants in our study are available in Supplementary Materials S8 Table. Gait signatures could be part of a set of multi-modal data to account for the diverse causal factors underlying each individual’s gait pattern (e.g., lesion neuroanatomy, medical confounding variables, musculoskeletal conditions, psychosocial variables, physiological contributors to gait, and environmental factors).”

4. Moreover, the authors are suggested to provide the medical details for lower-limb joints of the stroke patients. Have the authors considered existing discrepancies in lower-limb joints such as fixed flexion deformities (FFD)? Such discrepancies might affect the gait signatures significantly.

The cohort of post-stroke participants in the current study were part of larger clinical trials in our co-author's laboratory. The paper’s scope did not include understanding physiological contributors to gait signatures- this motivates future studies. However, to address the reviewer’s concern, as stated in our response above, we now include a couple statements in our revised Discussion lines 746-754 section pointing to the need for correlating clinical measures of strength, range of motion, and sensorimotor impairment with the RNN model results and acknowledges that individual differences in anthropometrics and joint function may affect walking in ways that would alter individual gait signatures. In future iterations of the method, it may be possible to add information about such deformities and demonstrate the effects on gait signatures. We have now provided a table in Supplementary Materials ‘S8 Table’ outlining the demographics and collected clinical characteristics of the stroke survivors in our dataset.

Lines 746-754: “Our study inclusion criteria exclude severe contractures or deformities that interfere with normal ambulation and in future work the gait signatures would need to be interpreted and correlated with clinical evaluation of strength, range of motion, sensorimotor impairment, and/or limb deformities. The demographic and clinical information of the stroke participants in our study are available in Supplementary Materials S8 Table. Gait signatures could be part of a set of multi-modal data to account for the diverse causal factors underlying each individual’s gait pattern (e.g., lesion neuroanatomy, medical confounding variables, musculoskeletal conditions, psychosocial variables, physiological contributors to gait, and environmental factors).”

| **Subject**  **ID** | **Sex** | **Age (yrs.)** | **Mo. Post-stroke** | **Paretic side** | **Lower-extremity Fugl-Meyer** | **Berg Balance Score** | **Self-Selected speed**  **(m/s)** | **Fastest speed (m/s)** |
| --- | --- | --- | --- | --- | --- | --- | --- | --- |
| ST1 | F | 74 | 24 | L | 26 | 54 | 0.45 | 0.70 |
| ST2 | F | 58 | 64 | L | 18 | 34 | 0.35 | 0.50 |
| ST3 | M | 59 | 46 | L | 23 | 42 | 0.37 | 0.47 |
| ST4 | M | 49 | 78 | R | 17 | 45 | 0.30 | 0.60 |
| ST5 | M | 59 | 15 | R | 23 | 56 | 0.55 | 0.80 |
| ST6 | M | 56 | 75 | L | 14 | 51 | 0.38 | 0.53 |
| ST7 | M | 36 | 35 | R | 20 | 56 | 0.45 | 0.80 |

**S8 Table: Stroke participant’s demographics and clinical scores**

5. There should be an algorithm (pseudo code) to highlight the computational approach involved in the development and implementation of RNN model. Moreover, the authors are suggested to provide a schematic representation of the proposed architecture with hyperparameters. The authors should justify the reason behind the selection of ADAM optimizer. Moreover, the details of the optimizer should be highlighted to improve the readability of the paper.

Thank you for this excellent suggestion. In addition to Figure S1, which demonstrates how we chose the number of nodes and the lookback length of the network, we have now added a graphical summary of the algorithm to the Supplemental Figures, S7 Figure.

**S7 Figure: Graphical summary and pseudo code of our gait signatures framework and algorithm.** A) **Training dataset**: Using the 6-dimensional gait trajectory, the inputs (green) were concurrent segments from the gait trajectory, each one 499-time steps long. The outputs (blue) were 1-shifted (in time) segments of inputs. B) **Model architecture**: Our model consisted of an input layer, a hidden layer composed of 512 LSTM units, and a 6-unit Dense output layer. C) **Stateful training**: The hidden state of an RNN at time t is a function of the input at time t and the hidden state at time t-1. The model starts with processing the first mini-batch, calculating a new hidden state at each t and predicting gait kinematics at time t+1 given kinematics data at t. At the end of the mini-batch processing, the model calculates MSE over the entire mini-batch to calculate error for backpropagation and to update model weights. The final hidden state h(t+L) is used as the initial hidden state for generating predictions for the next mini batch (L is the temporal length of each mini-batch). The hidden state is initialized as zero before processing the first mini-batch.

Thank you for the comment on the justification of using the ADAM optimizer. There are many optimization algorithms for performing stochastic gradient descent. We started model development using ADAM optimizer, a popular choice in deep learning as it is well documented, fast, has a small memory footprint, and is well suited for large-parameter deep learning models (Kingma et al. 2014). We found that we were able to train the model sufficiently well as our training and validation losses are significantly small (Figure 1 supplement 2). The effect of other adaptive SGD optimizers would be marginal (Zaman et al. 2021) and wouldn't affect our study. We included text in the Methods section Lines 891-894 to justify the selection of the ADAM optimizer and answer why the Adam optimizer was used as follows:

Lines 891-894: “Mean squared error was used as the LSTM loss function and ADAM as the optimization algorithm because it is fast, has a small memory footprint and is well suited for large-parameter deep learning models (Kingma et al. 2014).”

6. (Line 212) The authors claim that the training and validation error converged for less than 0.75 deg. They are suggested to provide the error convergence plot in addition to S2 Figure.

Thank you for your comment, our model was trained using the ‘mean squared error (mse)’ loss function. The training and validation error of the model converged to < 0.03 degrees^2^. We have updated the text in line 217-219 to reflect this.

Lines 217-219: “The model was trained using the ‘mean squared error’ (mse) loss function until training and validation error converged and stabilized around the same point (< 0.03 degrees^2^).”

7. (Line 246) The authors claim that six principal components (PCs) explained 77% of the variance in the gait dynamics. However, in view of S4 Figure, six PCs are able to explain ~71% of the gait variance. The authors are suggested to justify the reason of this discrepancy in the text.

We thank the authors for finding this discrepancy in our text. The figure S4 is accurate, and the six PCs can explain ~72% of the gait variance. We have updated the text in line 255 to correct this typo.

8. This study have focused on a specific gait pathologies (cortical or subcortical ischemic stroke) and only for seven patients, which may limit the applicability of the findings to other gait pathologies. This limitation can not be ignored by just mentioning in the discussion. Additionally, the study have not accounted for confounding variables, such as medical conditions or environmental factors, which could affect gait patterns.

We recognize that our sample size is limited, and that further work is needed with a larger set of stroke survivors and associated clinical metrics. Here we focus on demonstrating the innovation, feasibility, and potential advantages of our RNN gait signature approach. We reiterate this point in our revised Discussion lines 803-805. Our aim was only to show proof of concept that our simple RNN model can capture gait dynamics in a small cohort of stroke survivors across different walking speeds, and to justify further development as well as larger-sample studies. Related to confounding variables related to other medical conditions, based on our study’s exclusion criteria for stroke survivors, individuals with neurological diagnoses other than stroke were excluded, as well as people with other medical or orthopedic conditions affecting walking. The reviewer makes a good point about other factors such as environmental, psychosocial, etc. That may affect gait patterns, but we would like to note that the objective of the current RNN model was to capture individual differences in gait dynamics, irrespective of the neural, biomechanical, or other causal factors contributing to an individual’s gait pattern. Consistent with the reviewer’s point, our revised Discussion lines 746-754 now includes statements emphasizing the need to scale this and similar approaches to incorporate multi-modal data that can encapsulate the diverse causal factors underlying each individual’s gait pattern. We also note that the fact that our model performs well despite the small sample size showcases its promise as a methodological approach, and lays foundation for important future studies not only on stroke but also other pathologies affecting gait. Also, the clinical implications of this merit more systematic evaluation in future studies.

Lines 746-754: “Our study inclusion criteria exclude severe contractures or deformities that interfere with normal ambulation and in future work the gait signatures would need to be interpreted and correlated with clinical evaluation of strength, range of motion, sensorimotor impairment, and/or limb deformities. The demographic and clinical information of the stroke participants in our study are available in Supplementary Materials S8 Table. Gait signatures could be part of a set of multi-modal data to account for the diverse causal factors underlying each individual’s gait pattern (e.g., lesion neuroanatomy, medical confounding variables, musculoskeletal conditions, psychosocial variables, physiological contributors to gait, and environmental factors).”

Lines 803-805: “Here we focus on demonstrating the innovation, feasibility, and potential advantages of our RNN gait signature approach, justifying the need and potential for further development by scaling to larger-sample studies.”

**Reviewer #2**: The paper proposes a data-driven approach to analyze individual gait dynamics of able-bodied individuals and of stroke survivors. The model is based on a simple one-layer LSTM network, used to predict the evolution of the pose of the subjects during locomotion at different speeds. The hidden state of the LSTM is used to create a reduced base on which phase-averaged LSTM activations can be projected. Such projection, defined "locomotion signature", is analyzed in the attempt to identify differences in the gait across subjects. The main findings are:

- Able bodied, high-functioning, and low-functioning stroke survivors more or less cluster together
- Able bodied gaits form a more packed cluster
- The variability across gaits of different individuals exceeds that within an individual (even at different speeds),

justifying the definition of "locomotion signature"
- Individual components of the locomotion signature are interpretable. Parts of the signature from different

individuals can be combined, to investigate specific locomotion deficits.
- The LSTM can be used as a generative model. The able-bodied locomotion can be generated accurately.

over many cycles, while that of stroke survivors is only accurate for one cycle, and then converges to that of able-bodied individuals.

In my opinion the paper is very well written and motivated. The exposition is clear and precise. The methods are adequate and the authors have good knowledge of the tools they use.

I have some questions and comments:

- I am surprised that the differences in the gait at different locomotion speeds are much smaller than the differences between the gait of different individuals. I speculate that this is caused by 2 facts: 1. the sample size is small. With 13 individuals I can imagine that each gait has some unique, distinguishable features. If they had considered, e.g., 1000 individuals, I am quite sure the differences would have not been visible. Therefore, does it make sense to speak about a signature, when we do not know whether individual gaits would emerge in a larger dataset? 2. I think the speed range is also small. The authors hint to this fact (lines 289-290). This is a strong limitation, as one of the main results is that the variability across individuals exceeds the variability across locomotion speeds. The full range of locomotion speeds should be considered to prove something like this.

We agree that our demonstration of changes in gait speed is not comprehensive and potentially limited by the small data set we used. However, the data that we do have illustrates that the space of even this small population is much larger than the within-individual changes we observe across gait speed. Given the much larger ~3x, 4x, 7x distances between individuals versus within individuals for each group of able-bodied, high functioning and low-functioning individuals respectively, we do not expect this finding to change qualitatively when adding a larger number of individuals and speeds. Note that the speed range in stroke survivors does encompass the range of their safe walking capacity, so we do not expect to obtain broader ranges of walking speeds in the stroke group. Collecting larger ranges of speeds in non-disabled adults could expand our understanding of the sensitivity of gait signatures to walking speed which we intend to do in the future work. Of course, with a much larger data set (1000’s of individuals), we do expect that the unpopulated areas (white space) of our plots will begin to fill up, revealing a continuum of individuals. Potentially some individuals would be dynamically similar to other individuals but each individual’s gait signatures are still expected to group together across a space that may be overlapping to another individual, but nevertheless much smaller that the space subtended by the entire population. We have now added lines 290-293 to the Results section to provide quantitative metrics of inter-individual vs. Intra-individual differences in each group. We reiterate in lines 305-306 that we are already capturing the full range of safe walking capacity in stroke survivors. And we have clarified our expected results had we had an ideal dataset in lines 316-319.

Lines 290-293: “The gait signatures of the individuals belonging to the able-bodied, high-functioning, and low functioning stroke survivor cohorts show 3x, 4x and 7x larger distances between individuals in the group versus within each individual 6 speed trials respectively.”

Lines 305-306: “However, the walking speeds in our post-stroke cohort spanned the full speed range of each participant’s safe walking capacity.”

Lines 316-319: “Although the low-functioning individuals in our sample are more dispersed than high-functioning individuals, we expect that the spaces between individuals represent a continuum of gait dynamics that would be filled given a larger sample size.”

- The gait signature alignment is part of the model selection. Maybe it does not have a strong impact, but I am not sure it is correct to include the point you want to prove (that the hidden state of the LSTM defines a locomotion signature) it the selection of the model to use?

Thank you for your comment. Our aim is to encode gait dynamics. Because a good representation of the dynamics can predict the time evolution of gait, we used gait signature alignment as a more rigorous evaluation of our model’s ability to encode dynamics. We believe that including gait signature alignment as a model selection criterion is not problematic with respect to our outcomes. Rather, we selected the model prior to subsequent analyses, and gait signature alignment only guarantees the best self-driven predictions (average across trials) among the models tested (Figure 7). Therefore, our model selection paradigm gives us the model that best represents the underlying dynamics across all participants and gait speeds. Using any of our alternative architectures would reduce the representativeness of gait signatures for each participant. While the training and validation loss evaluate single timestep goodness-of-fit, it does not evaluate a model's ability to predict the time-evolution of gait so this additional criterion ensures that we have a model that can fulfil our aim of encoding gait dynamics. Once we have a model that can both fit the data well and predict the time-evolution of gait, the time-varying hidden states of the model (parameters) that encode how an individual’s kinematics evolves over time (dynamics) are used to generate our gait signatures. We have now clarified this point in the text lines 206-212.

Lines 206-212: “The model architecture was selected based on two criteria: 1) minimizing model training and validation loss during model fitting, and 2) maximizing the similarity of short-time (single stride) and long-time (multiple strides) self-driven model predictions (termed: gait signature alignment) post model training (S1 Figure). By implementing these two model selection criteria we ensure 1) a high goodness-of-fit (model that best represents the underlying dynamics across all participants and gait speeds) and 2) the model is capable of predicting the time-evolution of gait (encode gait dynamics).”

- It is briefly mentioned that gait signatures perform similarly to 26 commonly used discrete variables (fig. S5). The authors should better clarify why the features derived from the LSTM state should be preferred to these variables. They perform identically in classifying gait groups and individuals.

This analysis was used to give confidence that our gait signatures perform just as well as the current state-of-the-art in classification between able-bodied and stroke groups and between different individuals. Discrete variables already do a good job classifying between able-bodied adults and stroke survivors, and numerous studies (Kim H et al., 2022, Wikström J et al., 2014, Kaczmarczyk K et al., 2009) have shown which variables tend to map to walking function/impairment. Gait signatures, derived from the LSTM latent state, eliminate the need to heuristically select discrete variables for classification. Rather, gait signature-based classification groups individuals holistically using information spanning the multiple joints over the entire gait cycle. Further, our approach requires only kinematic measurements, making it faster and less resource-demanding than classification using discrete metrics: we achieve the same level of classification as discrete metrics that require force plates and inverse-dynamics analysis. Therefore, gait signatures enable classification with less equipment, computation, and subject-specific anthropometric measurements. We have now added the following clarification to the text in lines 322-326

Lines 322-326: “Discrete variables are already sufficient to classify between able-bodied and stroke gaits, with numerous studies identifying key variables that map to function/impairment [69–71]. With Gait signatures, we achieve the same level of classification without needing to hand-pick discrete variables or to use force plates or inverse-dynamics analyses that would require more equipment, computation, and subject-specific anthropometry for each observation.”

- There are some further clusters in the plots: there seem to be 2 able bodied clusters, almost as far from each other as the stroke survivors. What are these differences due to?

The major difference we found in able-bodied dynamics is in the PC3 which we believe encodes estimated postural differences or artifacts of data collection and processing. We do not have enough information about these individuals' history and lifestyle, but we will aim to collect it in the future. Due to our limited sample size of 5 AB individuals, we do see two apparent separate clusters of AB individuals on the left side of the plot, we believe that the white space represents unpopulated areas of the map and with more able-bodied data we will see that white space filled with more able-bodied dynamics representation. Despite able-bodied dynamics being very stereotypical, there still exists inherent variability in their gait dynamics – thus, the separation between individuals however their differences in gait dynamics are indeed smaller than the differences between gait groups (able-bodied and stroke) thus they occupy the left region of the map. Our future work will investigate a larger able-bodied cohort to quantify the variability in healthy dynamics and determine whether different clusters of dynamics do emerge.

- Fig. 3: not all low-functioning subclusters really look further from the able-bodied than the high-functioning ones. Overall, I doubt that any clustering algorithm would find the able-bodied, high-functioning and low- functioning clusters.

We agree with the reviewer that the low-functioning group does NOT form an obvious cluster, but rather could form multiple clusters or even a continuum of differences in gait. The low-functioning group has the most obvious differences, consistent with the fact that prior work has previously identified multiple subgroups of stroke survivors based on multiple types of impairments in muscle coordination which all lead to slow gait speed (Clark et al., 2010). This point is in fact a motivator of the current approach to find more holistic ways to separate out individuals who walk slowly for different reasons, and that may require different treatments. We also contend that there are variations within the able-bodied group that may also be explained by factors such as prior exercise and sports-training history, injury, disease, etc. We find this potential an exciting future application of the gait signatures approach, however, a much larger data set and more comprehensive information about participants will be required.

We have added the following text to the revised manuscript:

Lines 309-311: “Despite the able-bodied dynamics being similar, there still exists inherent variability in their gait dynamics that may be explained by factors such as prior exercise and sports-training history, injury, disease, etc.”

Lines 316-319: “Although the low-functioning individuals in our sample are more dispersed than high-functioning individuals, we expect that the spaces between individuals represent a continuum of gait dynamics that would be filled given a larger sample size.”

- Retraining the LSTM on any different dataset (or even just with different initialization) would lead a different basis vector, meaning that the analysis of the PCs has to be done for every model. Do they propose that the basis they found in this paper is used also in future studies? Or how do we do in practice?

This study provides only proof-of-concept for the gait signatures ideas. We do not believe that our limited dataset is comprehensive enough to form a reference model. The reviewer is correct that retraining the LSTM on different data and initializations would lead to a different basis vector. Ideally for practice, the goal is to have a large enough dataset to develop a more comprehensive set of basis vectors by which any new data coming in could be mapped into without re-rerunning a new model. Note that a large number of individuals of the same gait group (control/healthy, different impairments) would need to be trained on this hypothetical model. Also, in practice leveraging synthetic human data (Open sim / other musculoskeletal models) in addition to real data could be included to account for any edge cases in impairment and demographics (height, weight) as it would be impossible (or would take a long time) to get the ideal real- world dataset encompassing each point of height, age, weight, impairment. We have now clarified this point in the discussion in lines 814-817.

Lines 814-817: “In practice, a more comprehensive data set would be needed within each gait group to train a model capable of capturing the full range of variability in gait dynamics. Short of having a massive data set, it may also be possible to leverage synthetic gait data from simulations to span the full range of feasible gait dynamics variations.”

- The analysis named "gait sculpting" is very original and interesting in my opinion

We thank the reviewer for this comment. We envision one day having a tool where we can manually tune the PCs in such a way to generate the ideal gait pattern for an individual and use this to prescribe gait training or determine whether it is even possible to alter an individual’s gait in certain ways.

- the section 547-557 is not fully clear to me. Have the authors tried to set as an objective of the LSTM a longer trajectory, instead of the next pose? This might improve a lot the generative power of the network.

Although training the model to predict a longer time horizon forces the network to learn long timescale structure, it hinders our ability to keep the model stateful (the LSTM maintains its internal state from one time step to the next while a batch is being processed, after which the internal state is maintained for the next batch) during training and to run the model forward as an autonomous dynamical system. Similar issues would arise if one were to set up the model as a sequence-to-sequence auto-encoder predicting further than the next step, where the interpretability of the hidden state dynamics of the encoder and decoder LSTMs would be lost. However, it is important to note that even though our current framework only predicts one time-step in the future, the LSTM layer remains stateful through each gait cycle, allowing the model to learn much longer timescales as seen when self-driving the network (Figure 7A). Specifically, our network is trained in a sequence-to-sequence and stateful manner (lines 869-871 and S7 Figure), meaning that when the inputs {x(1), …, x(T)} are provided, the model predicts values {x'(2), … , x'(T+1)} causally (e.g., x'(2) only depends on the initial condition and x(1), x'(3) depends on x(1) and x(2), etc.). Granted, this is not to say that potential improvements are impossible, but rather that our model accounts for multiple time shifted relationships across gait that ultimately learns the dynamical system responsible for multiple joints, across gait phases, speed trials and individuals capable of predicting at least a future gait cycle of data. We have added text to the Methods section lines 1031-1034 to clarify how our training methodology generates longer timescale structure.

Lines 1031-1034: “However, it is important to note that even though our current framework only predicts one time-step in the future, the LSTM layer remains stateful through each gait cycle, allowing the model to learn much longer timescales as seen when self-driving the network (S7 Figure).”

The reviewer references the section 547-557 describing Figure 7 below not being fully clear:

To improve the clarity of this section we have reworded this text.

Lines 576-588: “To verify that the model was not generating a gait cycle prediction entirely by chance, we calculated the Euclidean distance between the kinematics of the predicted (self-driven) gait cycle and the kinematics from each of the measured gait cycles. We computed the distribution of distances between each predicted gait cycle with all other gait cycles from the same individual (Figure 7B, purple bars). We then compared the predicted gait cycle to the target gait cycle (Fig 7B, red bars). In the able-bodied individual, the predicted gait cycles are more similar to the target gait cycle (Fig 7B, i, red bar) than 79% of all gait cycles. However, in the stroke survivor, 60% of other gait cycles were more similar to the predicted gait cycle than the target gait cycle (Fig 7B, ii, red bar). This suggests that the model is less able to accurately predict future kinematics in stroke gait. Note, to calculate the Euclidean distances between the gait cycles, we need to normalize the period of each gait cycle to the period of the self-driven cycle. To avoid the potential of bias due to this normalization, we also performed a comparison using a metric that was not manipulated in time – gait cycle duration.”

**Minor reviewer comments:**

104 missing comma? - corrected
297 missing ) - corrected
299 "similarly to a when"? - corrected

572-573 strange sentence: “The potential of the gait dynamics model as a predictive tool provides a glimpse into the potential future application of our approach for predicting the effects of gait perturbations, technologies, and treatments, thus reducing time, cost, and participant burden while facilitating the development of more effective strategies to improve gait.”

We have now rephrased this sentence.

Lines 601-606: “Our model provides a foundation for a sample-specific gait dynamics model to predict the effects of environmental perturbations, assistive devices, and treatments without extensive experimental sessions. In-silico predictions using the gait dynamics model may thus reduce the experimental time, cost, and participant burden required for personalized gait characterization, treatment personalization, and device design.”

660-662 not clear that this result actually follows from the study: “Our data-driven gait dynamics model phenomenologically captures the net effects of both neural and mechanical constraints on gait and can further contribute to an understanding of normal and impaired gait.”

We agree with the reviewer that this result has not explicitly been tested or proved in our study and as such we have removed this claim from our revised manuscript.

737 strong title given the quality of the future predictions: “Gait signatures enable prediction of future kinematics”

Thank you for your comment. We have added a new analysis and supplementary figure (S6 Figure) which shows that gait signatures are capable of predicting (self-driven) a future gait cycle of multi-joint kinematics better than arbitrary gait cycle patterns and are indeed learning individual-specific gait patterns in both able-bodied and stroke survivors. However, we have updated the title in line 774 to read: “Gait signatures have potential to predict future kinematics”

752 extra full stop - corrected

**References**

Clark DJ, Ting LH, Zajac FE, Neptune RR, Kautz SA. Merging of healthy motor modules predicts reduced locomotor performance and muscle coordination complexity post-stroke. J Neurophysiol. 2010 Feb;103(2):844–57

Kaczmarczyk K, Wit A, Krawczyk M, Zaborski J. Gait classification in post-stroke patients using artificial neural networks. Gait & Posture. 2009 Aug 1;30(2):207–10.

Kim H, Kim YH, Kim SJ, Choi MT. Pathological gait clustering in post-stroke patients using motion capture data. Gait & Posture. 2022 May 1;94:210–6.

Kingma DP, Ba J. Adam: A Method for Stochastic Optimization. ArXiv. 2017

Wikström J, Georgoulas G, Moutsopoulos T, Seferiadis A. Intelligent data analysis of instrumented gait data in stroke patients—A systematic review. Computers in Biology and Medicine. 2014 Aug 1;51:61–72.

Zaman SM, Hasan MdM, Sakline RI, Das D, Alam MdA. A Comparative Analysis of Optimizers in Recurrent Neural Networks for Text Classification. IEEE Asia-Pacific Conference on Computer Science and Data Engineering (CSDE). 2021. p. 1–6.
